# A 13-gene signature to predict the prognosis and immunotherapy responses of lung squamous cell carcinoma

**DOI:** 10.1038/s41598-022-17735-6

**Published:** 2022-08-11

**Authors:** Qin Yang, Han Gong, Jing Liu, Mao Ye, Wen Zou, Hui Li

**Affiliations:** 1 Department of Oncology, the Second Xiangya Hospital of Central South University, Molecular Biology Research Center and Center for Medical Genetics, School of Life Sciences, Central South University, Changsha, 410000 Hunan China; 2 School of Medical Technology, Shao Yang University, Shaoyang, 422000 Hunan China; 3 Molecular Science and Biomedicine Laboratory, State Key Laboratory for Chemo/Biosensing and Chemometrics, College of Biology, College of Chemistry and Chemical Engineering, Collaborative Innovation Center for Chemistry and Molecular Medicine, Hunan University, Changsha, 410082 Hunan China; 4 Hunan Province Key Laboratory of Basic and Applied Hematology, Central South University, Changsha, 410011 China

**Keywords:** Non-small-cell lung cancer, Computational biology and bioinformatics

## Abstract

Lung squamous cell carcinoma (LUSC) comprises 20–30% of all lung cancers. Immunotherapy has significantly improved the prognosis of LUSC patients; however, only a small subset of patients responds to the treatment. Therefore, we aimed to develop a novel multi-gene signature associated with the immune phenotype of the tumor microenvironment for LUSC prognosis prediction. We stratified the LUSC patients from The Cancer Genome Atlas dataset into hot and cold tumor according to a combination of infiltration status of immune cells and PD-L1 expression level. Kaplan–Meier analysis showed that hot tumors were associated with shorter overall survival (OS). Enrichment analyses of differentially expressed genes (DEGs) between the hot and cold tumors suggested that hot tumors potentially have a higher immune response ratio to immunotherapy than cold tumors. Subsequently, hub genes based on the DEGs were identified and protein–protein interactions were constructed. Finally, we established an immune-related 13-gene signature based on the hub genes using the least absolute shrinkage and selection operator feature selection and multivariate cox regression analysis. This gene signature divided LUSC patients into high-risk and low-risk groups and the former inclined worse OS than the latter. Multivariate cox proportional hazard regression analysis showed that the risk model constructed by the 13 prognostic genes was an independent risk factor for prognosis. Receiver operating characteristic curve analysis showed a moderate predictive accuracy for 1-, 3- and 5-year OS. The 13-gene signature also performed well in four external cohorts (three LUSC and one melanoma cohorts) from Gene Expression Omnibus. Overall, in this study, we established a reliable immune-related 13-gene signature that can stratify and predict the prognosis of LUSC patients, which might serve clinical use of immunotherapy.

## Introduction

Despite a recent decline in the incidence and death rate, lung cancer is still the world’s leading cause of cancer death^1^. Over 80% of lung cancer patients are diagnosed with non-small cell lung cancer (NSCLC). Lung squamous cell carcinoma (LUSC) represents the second main NSCLC histotype^2^ and is particularly challenging to treat because of the highly heterogeneous nature and wide range of mutations present^3^. Immune checkpoint molecules (ICMs) are key regulators in maintaining immune homeostasis. Modulating ICMs expression (such as upregulating PD-L1 expression) is a normal strategy for cancer cells to escape from host immunity^4,5^. Immune checkpoint inhibitors (ICIs) are blocking-antibodies targeted to ICMs. The ICIs-based immunotherapy has revolutionized the standard care of patients with LUSC, prolonged overall survival (OS) recently. But a large portion of patients still does not experience tumor shrinkage or extended survival^2^. The current major clinical determinants of LUSC prognosis are traditional AJCC/UICC-TNM stratification systems; however, various outcomes of LUSC patients with similar AJCC/UICC-TNM features indicate that new reliable prognostic markers with higher sensitivity and accuracy are in need. Reliable signatures can evaluate benefits from immunotherapies and conduct new biomarker-directed immunotherapies for LUSC patients; however, there are no definitive biomarkers for predicting the immunotherapy response of patients at present.

The tumor is not only an accumulation of neoplastic cells, but constitutes a tumor microenvironment (TME). The composition of the TME differs across patients with the same kind of cancer, which has been demonstrated to be a major determinant of tumor characteristics and patient outcomes^6^. Recently, some studies have showed that the immune fraction of the TME has prognostic value in cancer. For example, the classification of tumors based on their immune phenotype of TME is used to explain the clinical response to ICIs-based immunotherapy. Immunologically hot tumor with a higher level of immune infiltration is prone to benefit from immunotherapy, while patients with immunologically cold tumors are more likely to be resistant to ICIs-based immunotherapy^7,8^. PD-L1 expression on tumor and immune cells was used to predict the response of patients receiving PD-1-based immunotherapy^9^. Cytotoxic T cells and helper T cells are potential prognostic factors following resection in NSCLC patients^10,11^. With the remarkable results achieved in immunotherapy, insight knowledge of the current guidelines for tumor classification, prognostic marker and subsequent treatment by analyzing TME composition has become a pressing necessity^12^.

This study aims to establish a prognostic multi-gene signature associated with the immune phenotype of TME for LUSC patients. We divide LUSC patients from The Cancer Genome Atlas (TCGA) dataset into two groups, hot and cold tumors, based on the immune infiltrate scores and PD-L1 expression level. To explore the underlying mechanism, we analyze the differential expression genes (DEGs) between the hot and cold tumors using enrichment analyses. Moreover, we identify hub genes based on the DEGs and constructed protein–protein interaction (PPI) network in hot and cold tumors. After that, a 13-gene signature based on the hub genes is developed and then, validated in multiple independent datasets across different platforms (TCGA and Gene Expression Omnibus (GEO)) and cancers (LUSC and melanoma). Overall, we are the first to specifically classify LUSC patients according to a combination of infiltration status of immune cells and PD-L1 expression. This study provides a reliable immune-related 13-gene signature that has significant implications for the prediction of outcomes for LUSC patients, which might facilitate the clinical use of immunotherapy.

## Materials and methods

### RNA sequencing data acquisition

Gene expression profile and corresponding clinical data of TCGA-LUSC were obtained from the “TCGA TARGET GTEx” cohort of UCSC XENA (http://xena.ucsc.edu/) and the Gene Expression Omnibus (GEO) (https://www.ncbi.nlm.nih.gov/geo/). The TCGA-LUSC dataset included 504 samples and the four databases (GSE30219, GSE12472, GSE157011 and GSE78220) from GEO included 606 samples. The clinical information of the TCGA-LUSC dataset was shown in Table 1.

**Table 1:** Clinical features of patients with lung squamous cell carcinoma in TCGA.

Clinical features	Count (%) (n = 504)
**Status**	
Alive	284 (56.3)
Dead	220 (43.7)
**Gender**	
Female	131 (26.0)
Male	373 (74.0)
**Age**	
≤ 65	190 (37.7)
> 65	305 (60.5)
Unknown	9 (1.8)
**Stage T**	
T1	114 (22.6)
T2	295 (58.5)
T3	71 (14.1)
T4	24 (4.8)
**Stage M**	
M0	414 (82.1)
M1	7 (1.4)
Unknown	83 (16.5)
**Stage N**	
N0	320 (63.5)
N1	133 (26.4)
N2	40 (7.9)
N3	5 (1.0)
Unknown	6 (1.2)
**Stage**	
Stage I	246 (48.8)
Stage II	165 (32.7)
Stage III	85 (16.9)
Stage IV	7 (1.4)
Unknown	1 (0.2)
**Neoplasm cancer status**	
Bronchial	10 (2.0)
L-lower	77 (15.3)
L-upper	137 (27.2)
R-lower	109 (21.6)
R-middle	18 (3.6)
R-upper	136 (27.0)
Unknown	17 (3.4)
**Smoking history**	
Current reformed smoker for < or = 15 years	252 (50.0)
Current reformed smoker for > 15 years	83 (16.5)
Current reformed smoker, duration not specified	5 (1.0)
Current smoker	134 (26.6)
Lifelong non-smoker	18 (3.6)
Unknown	12 (2.4)

The data collection and processing of this research complied with data policy of TCGA and GEO to protect human subjects. All experiments were performed in accordance with relevant guidelines and regulations.

### Identification of hot and cold tumors

ImmuneCellAI (http://bioinfo.life.hust.edu.cn/web/ImmuCellAI/) was used to estimate the abundance of 24 immune cells and infiltration scores from gene expression dataset^13^. The Immune infiltration level was used to combine with the PD-L1 expression level for dividing LUSC tumors into two groups: hot and cold tumors^14^. The hot tumors were of both the top 50% immune infiltration and the top 50% PD-L1 expression levels, while the others were defined as cold tumors. The Kaplan–Meier curve was performed for OS analysis of the hot versus cold tumors. OS was the length of time from the date of diagnosis or the start of treatment for a disease, such as cancer, that patients diagnosed with the disease were still alive.

Furthermore, our definition of the hot and cold tumors was validated by the expression of ICMs, ESTIMATE and CIBERSORT analyses. ICMs such as PD-L1 were used to predict clinical outcomes with ICIs-based immunotherapy^15^. ESTIMATE was used to calculate the immune score, stromal score, ESTIMATE score and tumor purity^16^. Stromal and immune scores were used to predict the level of infiltrating stromal and immune cells. ESTIMATE score based on the immune score and stromal scores were further used to infer tumor purity in tumor tissue^16^. CIBERSORT algorithm was performed to calculate the abundance of 22 types of immune cell subsets in each sample^17^.

### Identification of differentially expressed genes (DEGs) and enrichment analysis

The R package “DESeq2”^18^ was applied to identify DEGs (p.adjust value < 0.05 and |log2FC|> 1) between the hot and cold tumors. Afterward, the DEGs were used to perform Gene Ontology (GO) and Kyoto Encyclopedia of Genes (KEGG) analyses by using the R package “clusterProfiler”^19^. All genes were arranged into a ranked list according to the fold change and then used to conduct a GSEA analysis. For the target set of GSEA analysis requirements, we obtained hallmark gene sets (h.all.v7.4.entrez.gmt) from Molecular Signatures Database (MSigDB). Pathways with p.adjust value < 0.05 were selected.

### Protein protein interaction (PPI) network and hub genes identification

To further investigate the interactions between the DEGs, a PPI network was constructed using the STRING (https://string-db.org/) database and interaction scores > 0.4 were considered statistically significant. The MCODE was a tool to detect densely connected regions in large protein–protein interaction networks that might represent molecular complexes^20^, which was used to extract key sub-networks and identify hub genes in the network. Enrichment analyses were performed on the hub genes. Subsequently, the CytoHubba^21^ was used to obtain the top 10 hub genes in hot and cold tumors, respectively. All parameters were default values and all the above networks were visualized in Cytoscape software (v3.8.2).

### Screening of prognostic multi-gene signature

The univariate Cox regression analysis was used to obtain prognostic genes in the hub genes. The prognostic 13-gene signature was established by the multivariate cox analysis and the least absolute shrinkage and selection operator (LASSO) analysis^22^. We evaluated the risk score of each patient by the below formula:

$$ {\text{Risk score}} = {\text{Coef}}_{{{\text{gene1}}}} \times {\text{Exp}}_{{{\text{gene1}}}} + {\text{Coef}}_{{{\text{gene2}}}} \times {\text{Exp}}_{{{\text{gene2}}}} + \cdots + {\text{Coef}}_{{{\text{gene13}}}} \times {\text{Exp}}_{{{\text{gene13}}}} . $$

The Kaplan–Meier survival curve was used validate the prognostic value of the 13-gene signature. Receiver operating characteristic (ROC) curve analysis was used to estimate the sensitivity and specificity of the 13-gene signature. Moreover, to demonstrate that the risk score was an independent prognostic factor, we conducted univariate and multivariate Cox regression analyses to examine the prognostic value of the risk score and other clinical indicators in the TCGA-LUSC patients.

### Statistical analysis

The Wilcoxon test was used to compare the differences in the proportion of immune cells, ICMs expression levels and ESTIMATE scores between the hot and cold tumors. For the OS analysis, the Kaplan–Meier curve and the two-sided log-rank test were performed. R package v4.0.2 was performed for all analyses and p < 0.05 was considered as statistical significance.

## Results

### Stratification of hot and cold tumors

**Figure 1:**
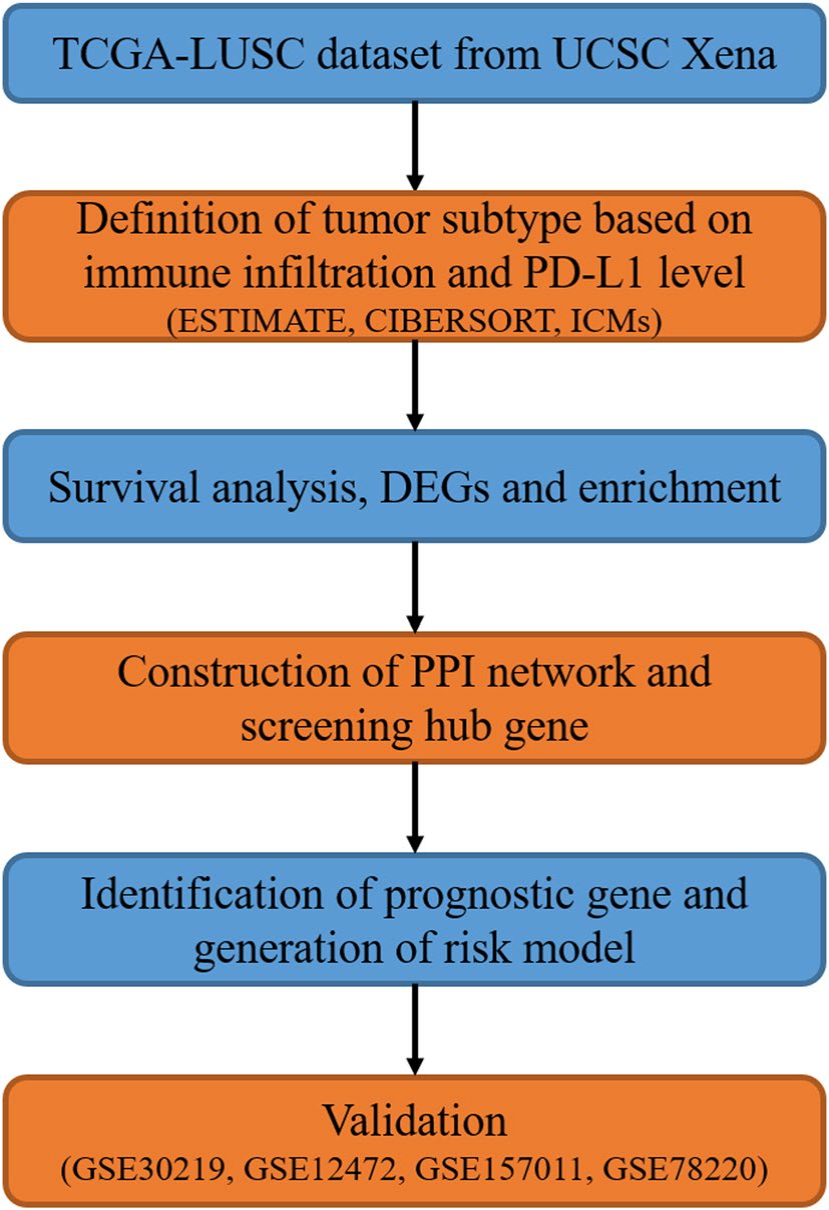
Flow chart of this study. The flow chart shows the strategy for developing and validating an immune-related 13-gene signature. This signature is constructed to predict immunotherapy responses and the overall survival of lung squamous cell carcinoma.

**Figure 2:**
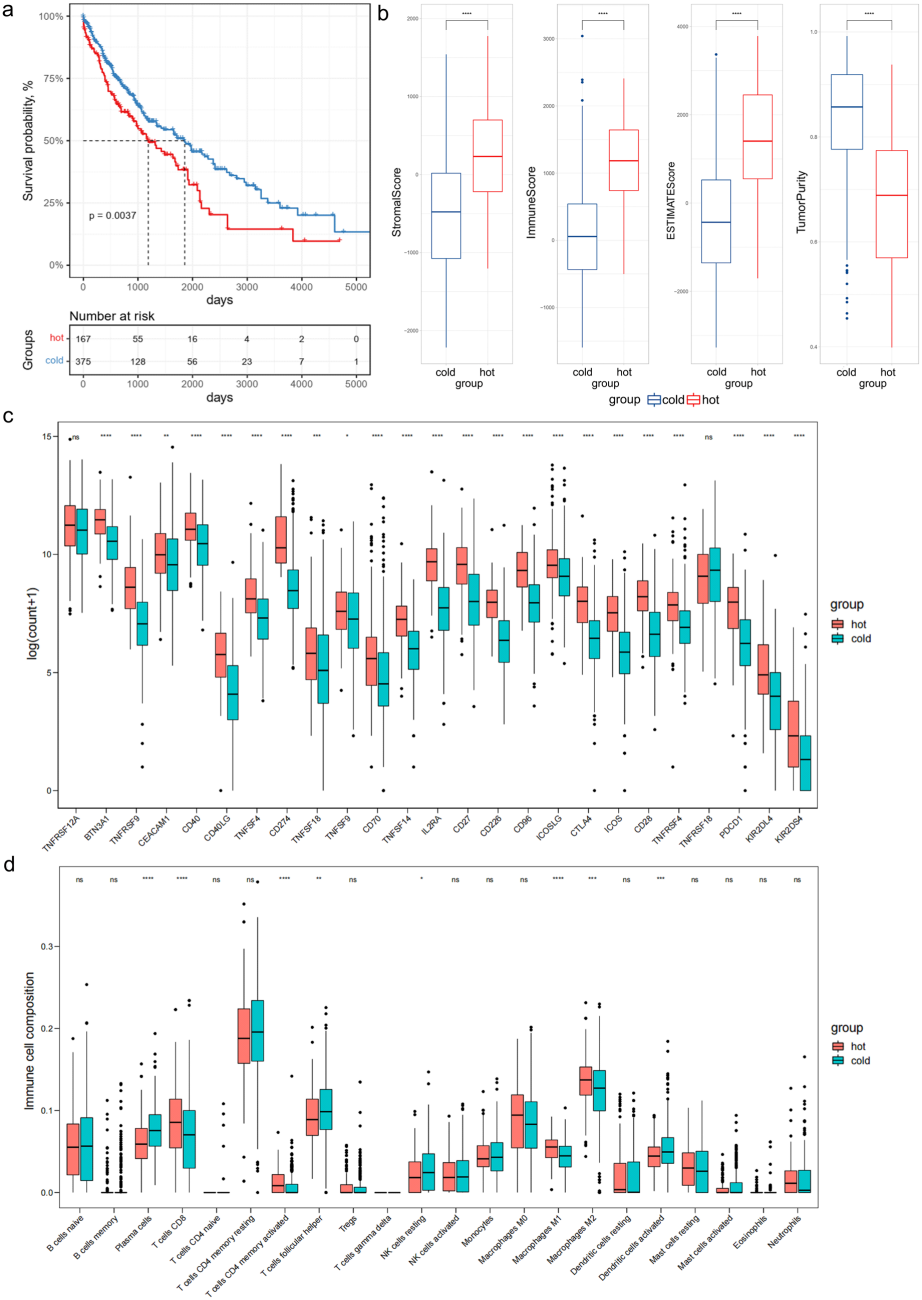
Stratification of hot and cold tumors. (**a**) The Kaplan–Meier curve shows the correlation between tumor stratification and overall survival. The p value is based on the log-rank test. (**b**) Immune score, stromal score, ESTIMATE score and tumor purity were obtained from ESTIMATE analysis in hot and cold tumors. (**c**) Expression of immune checkpoint molecules in hot and cold tumors. (**d**) Boxplot shows 22 immune cells infiltration obtained from CIBERSORT analysis in hot and cold tumors. ∗p < 0.05, ∗∗p < 0.01, ∗∗∗p < 0.001, ∗∗∗∗p < 0.0001, *hot* hot tumors, *cold* cold tumors.

To construct a multi-gene signature for predicting OS of LUSC patients, we designed and processed our study as shown in the flow chart (Fig. 1). Hot tumors are supposed to have a relatively higher immune infiltration and are thus more likely to respond to immunotherapy compared with cold tumors^7,8^. PD-L1 is a co-inhibitory ICM that contributes to the immune escape of cancer cells^5^. LUSC patients with an upregulated PD-L1 expression are more likely to benefit from immunotherapy^23^. Five hundred and four LUSC-TCGA tumor samples (Table 1) were divided into two groups: hot and cold tumors, when a combination of immune infiltration scores and the PD-L1 expression level was used as a cutoff. Tumors responding to immunotherapy had the top 50% immune infiltrates scores and the top 50% PD-L1 expression were referred to as ‘hot tumors’, whereas the rest tumors were ‘cold tumors’. Kaplan–Meier analysis demonstrated that patients with hot tumors had a significantly shorter OS than patients with cold tumors (Fig. 2a). We subsequently compared immune cell infiltration levels between the hot and cold tumors using ESTIMATE (Fig. 2b), the expression level of co-stimulatory ICMs and CIBERSORT (Fig. 2c,d). Most of the co-stimulatory ICMs were upregulated in hot tumors (Fig. 2c). Immune cells were more infiltrated in hot tumors than cold tumors (Fig. 2b–d). These results together indicated that hot tumors were more likely to respond to immunotherapy than cold tumors.

### Enrichment analyses of the DEGs

**Figure 3:**
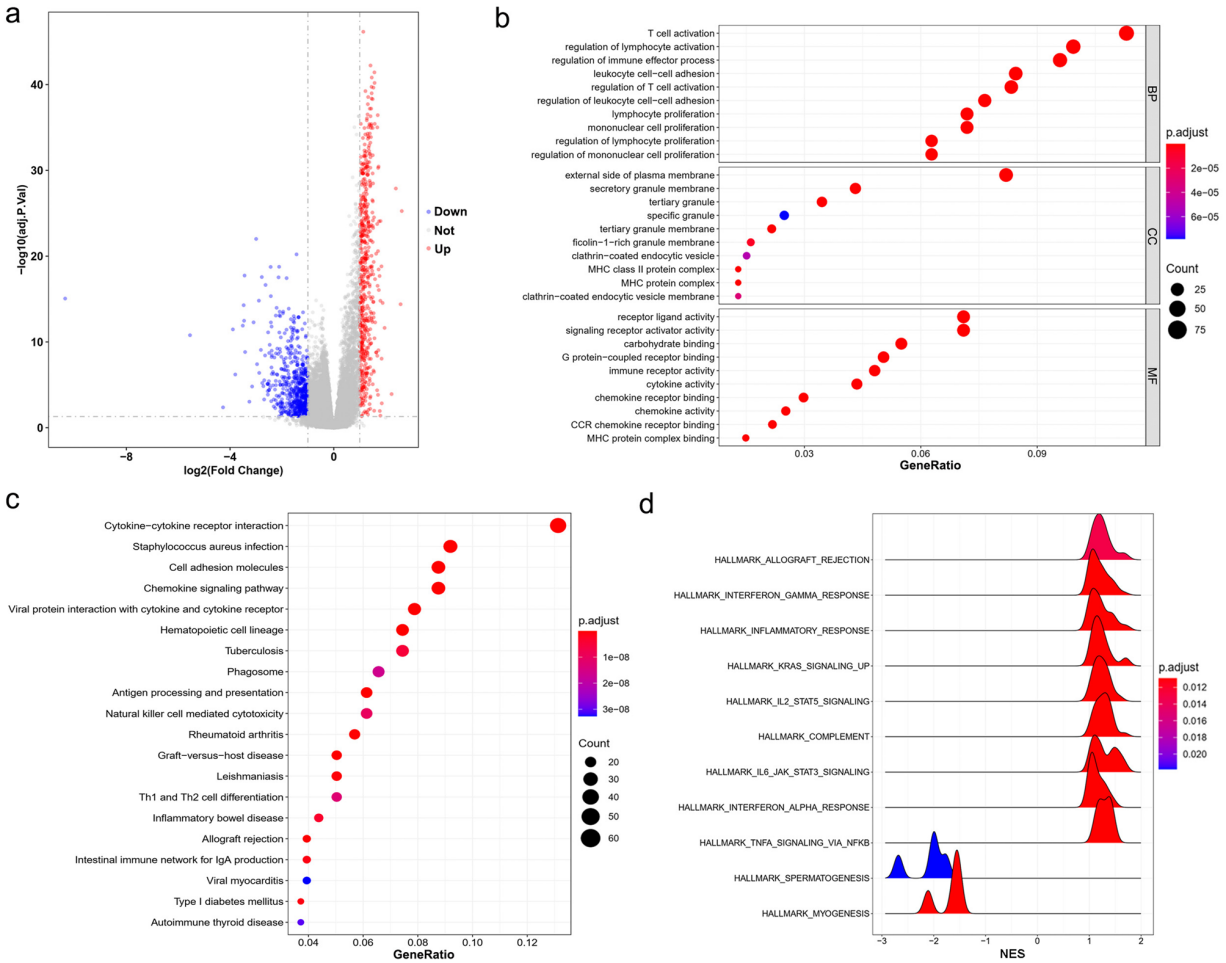
Enrichment analyses of differentially expressed genes (DEGs) between hot and cold tumors. (**a**) Volcano plot of the DEGs in the hot and cold tumors. Red plots (UP) represent significantly upregulated DEGs. Blue plots (Down) represent significantly downregulated DEGs. Grey plots (Not) represent DEGs with no significant difference. The vertical dashed line represents |log2FC|> 1 (fold change > 2) and the horizontal dashed line represents p.adjust value = 0.05. P.adjust value < 0.05 is considered significant. (**b**) Gene Ontology enrichment analysis of the top 10 enrichments for biological process, cellular component and molecular function. Enrichment levels are showed as a continuous variable from blue to red color. The bluer color represents the higher p.adjust value, and the redder color represents the lower p.adjust value. Enrichment counts are showed by dot sizes. The bigger size represents the higher enrichment count and the smaller size represents the lower enrichment count. (**c**) Kyoto Encyclopedia of Genes and Genomes analysis^24–26^. (**d**) Gene Set Enrichment Analysis is showed by normalized enrichment scores (NES) on X-axis and enriched pathways on Y-axis. The NES < 0 represents pathways enriched in cold tumors while the NES > 0 represents pathways enriched in hot tumors.

1203 DEGs (including 564 upregulated DEGs and 639 downregulated DEGs) were identified in the hot compared with cold tumors (Fig. 3a). To gain a functional understanding of the DEGs, we conducted GO (Fig. 3b) and KEGG (Fig. 3c) analyses on the 1203 DEGs. We also performed a GSEA analysis (Fig. 3d) based on the rank information of all genes. The GO biological process (BP) mainly included the proliferation and regulation of multi-immune cells (Fig. 3b). The most abundant GO molecular function (MF) was immune receptor activity (Fig. 3b). GO cellular component (CC) was enriched in ‘neuroactive ligand-receptor interaction’ and ‘metabolism of xenobiotics by cytochrome P450’ (Fig. 3b). Pathway enrichment analysis regarding KEGG focused on ‘cytokine-cytokine receptor interaction’, ‘cytokine signaling pathway’, ‘antigen processing and presentation’ and ‘natural killer cell-mediated cytotoxicity’ (Fig. 3c). The GESA was enriched in the hallmark gene sets of ‘interferon α/γ response’, ‘inflammatory response’, ‘IL5-STAT5 signaling’, ‘IL6-STAT3 signaling’ and ‘TNF-α signaling via NF-KB’ (Fig. 3d).

### Hub genes and protein–protein interactions (PPIs)

**Figure 4:**
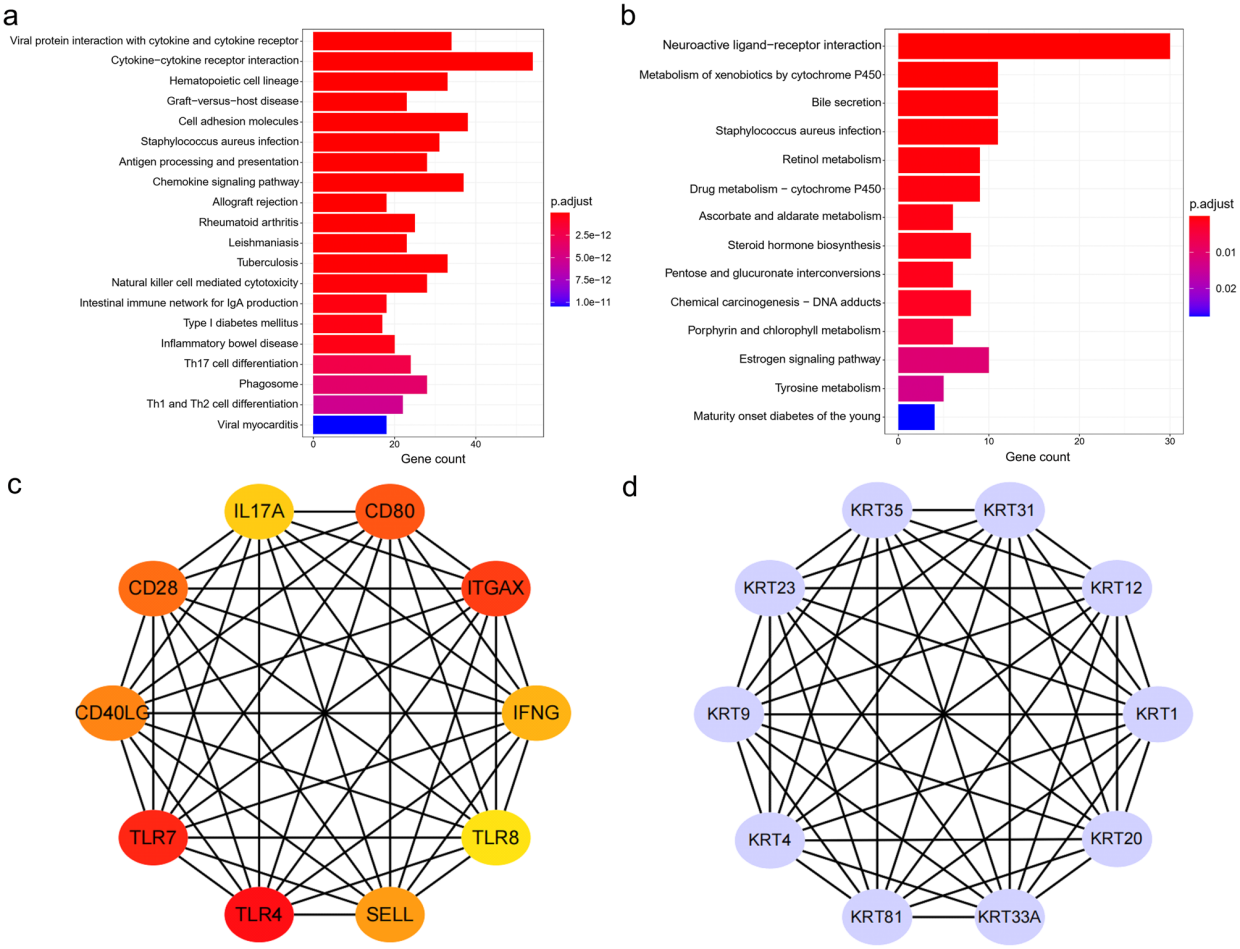
Kyoto Encyclopedia of Genes and Genomes (KEGG) enrichment analysis^24–26^ and protein–protein interaction (PPI) network. (**a**) KEGG analysis of the upregulated genes for hot tumors. Enrichment levels are showed as a continuous variable from blue to red color. The bluer color represents the higher p.adjust value, and the redder color represents the lower p.adjust value. P.adjust value < 0.05 is considered significant. (**b**) KEGG analysis of upregulated genes for cold tumors. (**c**) PPI network of top 10 hub genes obtained from CytoHubba analyses in hot tumors. Gene rankings are shown as colors. The redder color represents the higher gene ranking, and the yellower color represents the lower gene ranking. (**d**) PPI network of top 10 genes obtained from CytoHubba analysis in the cold tumors. The 10 genes are the same as the gene ranking.

To further explore the functions of DEGs, we conducted a KEGG analysis in hot and cold tumors, respectively. When compared with the cold tumor, the upregulated DEGs in the hot tumors were mainly enriched in immune-related pathways (Fig. 4a) and the downregulated DEGs in the hot tumors were mainly enriched in metabolic pathways (Fig. 4b). To further explore the interaction between the DEGs, we constructed PPI networks by STRING in hot and cold tumors, respectively. Afterward, 337 hub genes based on the 1203 DEGs were identified through MCODE in Cytoscape software. The hub genes have relatively higher intro module connectivity and gene significance than the other genes and play key roles in pathways in the co-expression network. The top 10 hub genes in hot and cold tumors were filtered into the PPI network (Fig. 4c,d). The common feature of the top hub genes (for instance, IL17A, CD28, CD80 and CD40LG) in hot tumors was that they were involved in the immune activation process directly or indirectly (Fig. 4c). The top hub genes in cold tumors were keratin (KRT) family members, which were not so closely related to immune responses (Fig. 4d).

### Identification of an immune-related 13-genes signature

**Figure 5:**
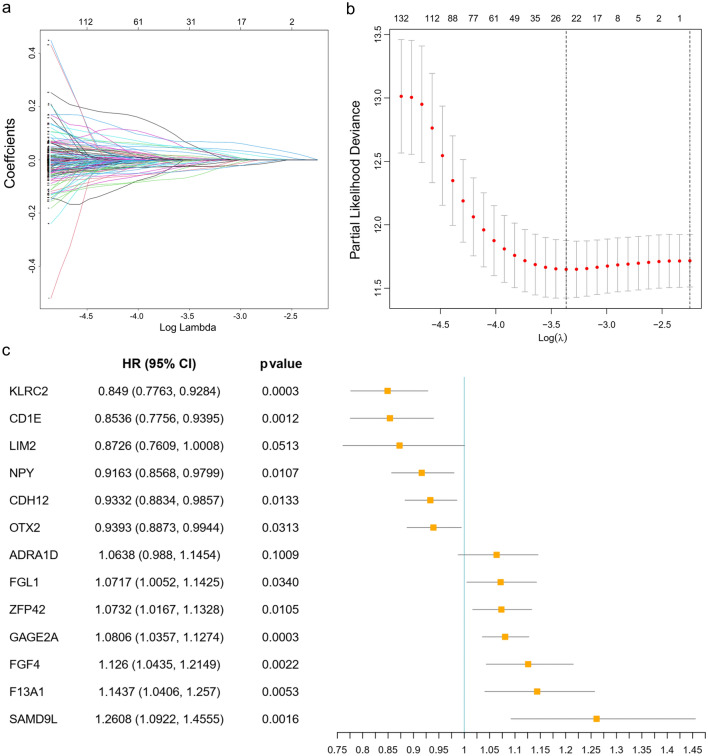
Identification of 13 prognostic genes in The Cancer Genome Atlas cohort. (**a**,**b**) Partial likelihood deviance coefficient profiles and Least absolute shrinkage and selection operator cox analysis of the hub genes. After 10 rounds of cross-validation, the optimal value of turning parameter is determined as 23. (**c**) Hazard ratios (HR) forest plot of the 13-gene signature constructed by the multivariate cox analysis. HR > 1 indicates that the gene is a risk factor. HR < 1 indicates that the gene is a protection factor.

To estimate the value of the 337 hub genes in predicting OS in LUSC, the TCGA-LUSC dataset was used as a training cohort. The univariate Cox regression analysis was used to obtain prognostic genes in the above hub genes. Subsequently, the LASSO and multivariate cox regression analyses were performed to identify prognostic genes with the strongest predicting ability in the training cohort (Fig. 5a,b). Finally, 13 prognostic genes (risk model) were identified (Fig. 5c) and the risk score was calculated by the following formula: risk score = (0.11865 × FGF4 expression) + (0.06922 × FGL1 expression) + (− 0.13624 × LIM2 expression) + ( − 0.08743 × NPY expression) + (0.13426 × F13A1 expression) + ( − 0.06918 × CDH12 expression) + ( − 0.15824 × CD1E expression) + ( − 0.06260 × OTX2 expression) + (0.06185 × ADRA1D expression) + (0.23177 × SAMD9L expression) + (0.07060 × ZFP42 expression) + (0.07751 × GAGE2A expression) + ( − 0.16373 × KLRC2 expression). Functions of the 13 prognostic genes were showed in Supplementary Table S1.

**Figure 6:**
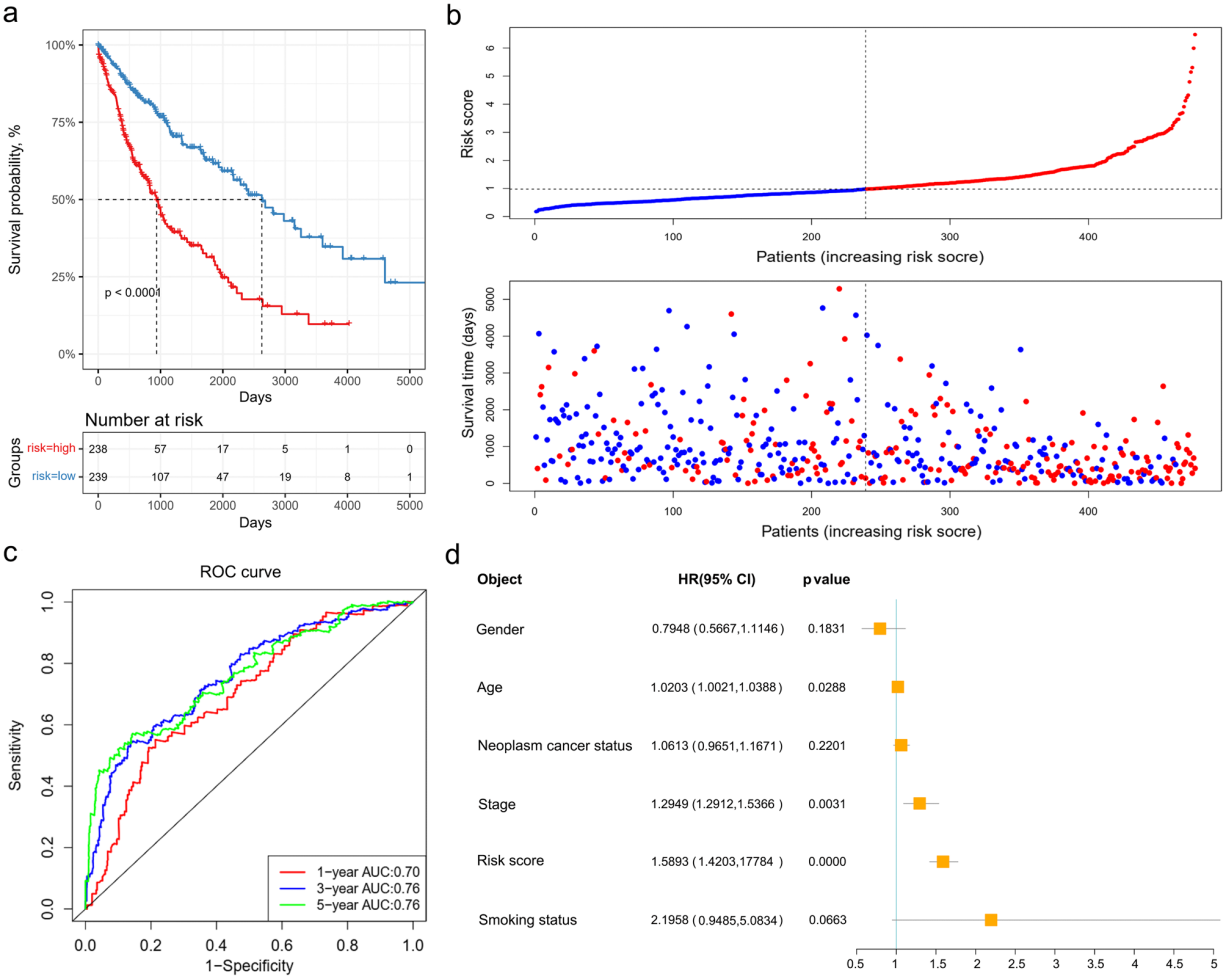
Validation of the 13-gene prognosis signature in The Cancer Genome Atlas cohort. (**a**) Kaplan–Meier curves of overall survival of the high- and low-risk groups. The p value is based on the log-rank test. (**b**) The distribution of risk scores, patient relapse status and survival time. In the upper panel, the red color represents the high-risk group and the blue color represents the low-risk group. In the lower panel, red dots represent dead patients and blue dots represent alive patients. (**c**) Receiver operating characteristic curve (ROC) for the 1-, 3-, and 5-year survival prediction using the 13-gene signature. *AUC* area under the ROC curve. (**d**) Multivariate cox analysis shows the hazard ratios of the 13-gene signature and other clinico-pathological factors with forest plots.

**Figure 7:**
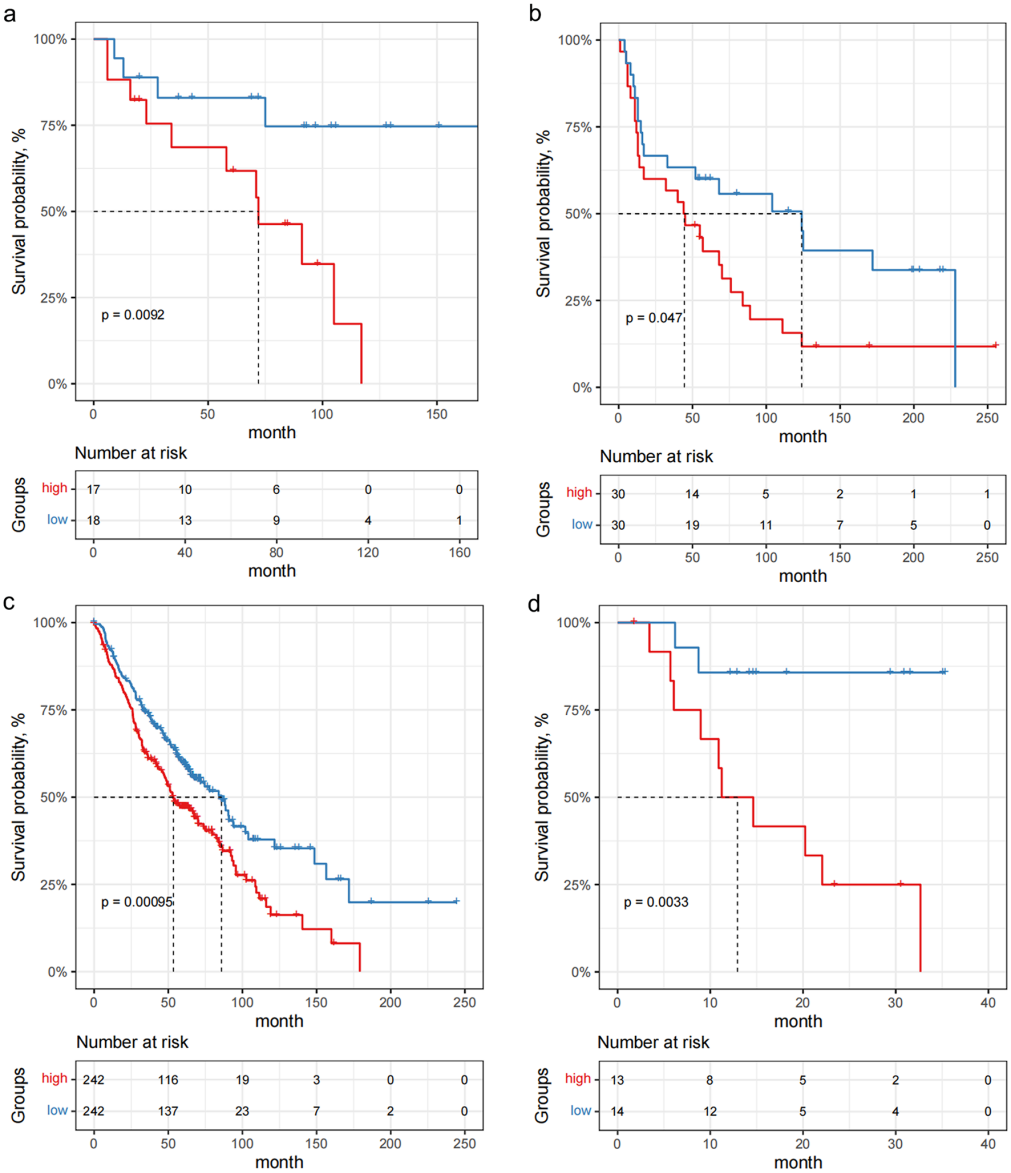
Re-sampling analyses in Gene Expression Omnibus datasets (GSE30219, GSE12472, GSE157011 and GSE78220) implicate the reliability of the 13-gene signature for predicting overall survival of LUSC patients. (**a**) Kaplan–Meier survival curves for overall survival in the lung squamous cell carcinoma (LUSC) immunotherapy dataset GSE12472. The p value is based on the log-rank test. (**b**) Kaplan–Meier survival curves for overall survival in the LUSC immunotherapy dataset GSE31210. The p value is based on the log-rank test. (**c**) Kaplan–Meier survival curves for overall survival in the LUSC immunotherapy dataset GSE157011. The p value is based on the log-rank test. (**d**) Kaplan–Meier survival curves for overall survival in the melanoma immunotherapy dataset GSE78220. The p value is based on the log-rank test.

By the median risk score, LUSC patients were divided into high- and low-risk groups and Kaplan–Meier curve showed that poor OS outcomes of LUSC patients were associated with the high-risk scores (Fig. 6a). According to the risk scores, the LUSC patients were ranked from left to right shown in the upper panel of Fig. 6b. The risk scores increased from left to right. OS distribution of each patient was shown in the lower panel of Fig. 6b where LUSC patients were ranked from left to right according to risk scores. A ROC curve was constructed to analyze the diagnostic accuracy of the 13-gene signature. It revealed that the 13-gene signature could serve as valuable biomarker for distinguishing between LUSC and control subjects (for 1-year, areas under the curve (AUCs) = 0.70; for 3-year, AUC = 0.76; for 5-year, AUC = 0.76) (Fig. 6c). To determine if the 13-gene signature was an independent prognostic marker, univariate and multivariate cox regression analyses were performed on the TCGA-LUSC dataset. The risk score of the 13-gene signature and other clinico-pathological factors, including gender, age, neoplasm cancer status, stage and smoking status were used as covariates in the cox regression analysis. We found a significant association between the 13-gene signature and OS in the TCGA dataset (HR = 1.5893, p < 0.0001). Our results showed that this 13‑gene signature was an independent risk factor for predicting the OS of LUSC patients (Fig. 6d). The detailed univariate and multivariate cox analyses of the 13-gene signature and other clinico-pathological factors were showed in Table 2. External four GEO-LUSC datasets (GSE30219, GSE12472, GSE157011 and GSE78220) were utilized to validate the prediction power of the 13-gene signature, of which GSE78220 is a melanoma immunotherapy dataset. In line with the results in the training cohort (TCGA dataset), the Kaplan–Meier curve indicated that the risk scores could distinguish the patients well in the GEO datasets (Fig. 7); LUSC patients with low-risk scores demonstrated a significantly longer OS in the three validation cohorts. Based on these results, the 13-gene signature performed well in predicting OS of LUSC patients and could potentially guide the clinical management.

**Table 2:** Univariate and multivariate cox regression analyses of the prognosis-related factors.

Variables	Univariate analysis			Multivariate analysis		
	HR	95% CI	p value	HR	95% CI	p value
Gender	0.824	0.824 (0.596, 1.140)	0.243	0.795	0.795 (0.567, 1.115)	0.183
Age	1.014	1.014 (0.997, 1.031)	0.097	1.020	1.020 (1.002, 1.039)	0.029
Neoplasm cancer status	1.082	1.082 (0.987, 1.187)	0.094	1.061	1.061 (0.965, 1.167)	0.220
Stage	1.297	1.297 (1.098, 1.53)	0.002	1.295	1.295 (1.091, 1.537)	0.003
Risk score	1.634	1.634 (1.467, 1.820)	3.96E−19	1.589	1.589 (1.420, 1.778)	6.67E−16
Smoking status	1.683	1.683 (0.743, 3.813)	0.212	2.196	2.196 (0.949, 5.083)	0.066

## Discussions

LUSC comprises about 20–30% of all lung cancers^27^. Its clinical outcome has been poor for the past decades, because of a limited treatment strategy. The situation has dramatically changed mainly with the clinical introduction of ICIs-based immunotherapy in recent years; however, it is quite clear that only a subgroup of LUSC patients achieves sustained benefits from ICIs-based immunotherapy. At present, various LUSC outcomes have been identified in patients with similar clinical and pathological features, suggesting that the current clinical prognostic factors used may be insufficient to consistently predict individual clinical outcomes. Predictive indicators are the most important to choose rational treatment. Identifying reliable prognostic markers with higher sensitivity and accuracy in LUSC is in urgent need. Molecular markers on tumor have been extensively investigated for prognosis and guidance of cancer therapy; much less for tumor-associated immune cells in TME. TME is an environment where tumors are considered as complex dynamic tissues with an important interplay of various cells including tumor-infiltrating immune cells, which is crucial for identifying effective biomarkers for predicting drug resistance and cancer progression^28^. Many studies have demonstrated that T cells are the major immune cells infiltrating tumors in TME and the degree of lymphocytic infiltration is positively associated with an absence of tumor metastases^29^. ICMs are indispensable for the full activation of T cells. PD-L1, as a major ICM, is expressed on the cell surface in tumor-associated immune cells and various cancer cells^5^. Although the expression of PD-L1 has been widely evaluated in ICI-based immunotherapies as a positive predictive marker, it is still an imperfect predictive biomarker^2,30^.

In our study, for the first time, LUSC patients distributed in hot and cold tumors were characterized by a combination of immune cell infiltration and PD-L1 expression associated with TME. Tumors responding to immunotherapy had a higher level of PD-L1 expression (top 50%) and immune infiltrates scores (top 50%) were referred to as ‘hot tumors’, whereas the rest of the tumors were ‘cold tumors’. Hot and cold tumors defined by our method predicted well in OS of LUSC patients. ESTIMATE, the expression level of co-stimulatory ICM, CIBERSORT and enrichment analyses all suggested that the hot tumors potentially had a higher immune response to immunotherapy than cold tumors, which further approved our stratification of tumors. Recently, this unofficial classification of tumors into two categories, ‘hot tumors’ and ‘cold tumors’, has been increasingly advocated. This immune-based, rather than tumor-based patient classification according to tumor immune infiltration, has shown a greater relative prognostic value than the traditional AJCC/UICC-TNM stratification system^12,31,32^. Different classifications of tumors represent various responses to immunotherapeutic options. Hot tumors are more likely to benefit from immunotherapy. Our stratification of LUSC patients contribute to dichotomizing tumors and can ultimately contribute to converting cold tumors to hot tumor.

Hot tumors showed remarked differences in hub genes profile from the cold tumors. We found that the top hub genes of hot tumors comprised many immune-related genes (for instance, IL17A, CD28, CD80 and CD40LG). Co-stimulatory ICMs CD28, CD80 and CD40LG are secondary signal molecules in the T lymphocyte activation, which activate patients’ anti-tumor immune responses, leading to increased efficacy of cancer immunotherapy^5^. CD28 is associated with an abundance of lymphocytes and longer OS in lung adenocarcinoma (LUAD)^33^. CD80 activates effector T cells via interacting with the receptors CD28 on the surface of the T cells. Upregulated CD80 predicts good prognosis in gastric adenocarcinoma^34^ and oral squamous cell carcinoma^35^. Expression of CD40LG in the tumor-free lymph node is positively related to a good prognosis in oral squamous cell carcinoma^35^. We also observed that all top hub genes in cold tumors were keratin family members (for instance, KRT20, KRT12 and KRT4). Keratins are expressed in highly specific patterns correlated to the epithelial type and stage of cellular differentiation. Characteristic expression patterns of keratins are also observed in cancers^36^. Moreover, keratins are diagnostic and prognostic markers in epithelial cancers. For example, downregulated hub gene KRT20 indicates poor patient outcomes in colorectal cancer^37^, pancreatic adenocarcinomas^38–40^ and gastric cancer^41^. Soluble keratins in the circulation of NSCLC patients carry prognostic significance and are used to monitor tumor load and disease progression in clinical practice^42,43^. Cold tumors are the most challenging to eradicate and are invariably associated with a poor prognosis. Our results on the top hub genes in the cold tumors suggested a critical role of keratins in immunotherapeutic resistance. In line with this result, one widely accepted role of keratins is a protector of mechanical stability and epithelial cell integrity under a variety of stressful conditions including death receptor activation and drugs^42,43^. Further studies are required to identify the mechanisms of keratins explaining this possible decreased susceptibility and identifying prognostic markers in immunotherapy of LUSC.

A gene signature predicting the prognosis of a large cohort of cancer patients is of great significance, because the gene expression can capture the influence of the changes of multiple genes at the same time and summarize the prognosis of multiple ‘conventional’ risk factors into one risk score^44,45^. Although gene expressions currently are not involved in the standard diagnosis of LUSC, it is proved to be a comprehensive tool for predicting outcomes in many cancers. For instance, a 3-gene signature has been proved to be a comprehensive tool for leukemia diagnosis and classification due to its high accuracy in all clinically relevant leukemia sub-entity predictions^44^. Zheng et al. have identified a 9-gene signature to predict OS in LUAD patients^46^. In this study, we applied multi-cox regression analysis and LASSO feature selection to screen a 13‑gene signature among 337 hub genes. In TCGA and three GEO cohorts validation, the 13-gene signature significantly stratified patients into high- vs low-risk groups in terms of OS and remained as an independent prognostic factor in multivariate analysis. Among the 13 prognostic genes, CD1E, KLRC2 and GAGE2A are more relevant to tumor immunity. CD1E is an MHC class I-like molecule that presents antigens to T cells and thus regulates T cells participation in the immune response. CD1E can predict the efficacy of immunotherapy in patients with nonmuscle-invasive bladder cancer^47^ and glioblastomas^48^. KLRC is expressed primarily in natural killer (NK) cells. Tumor infiltration of NK cells is correlated with the prolonged survival of cancer patients. Either acute exercise or in vitro expansion of KLRC+/NKG2A− NK cells can enhance the anti-tumor cytotoxicity of NK cells for immunotherapy^49^. In ovarian cancer, tumor-specific antigen GAGE2A can be used as an indicator for early diagnosis, efficacy evaluation and prognostic determination^50^.

In conclusion, for the first time, by dividing tumors into hot and cold tumors according to a combination of their immune infiltration and PD-L1 expression, this study proposed an immune-based rather than a tumor-based classification specifically for LUSC. Moreover, an immune-related 13-gene prognostic signature was developed and validated for prognosis prediction in LUSC through multi-step bioinformatics. This signature was strongly associated with OS in LUSC patients and might serve as a potential prognostic biomarker for clinical use of immunotherapy in the future. Prospective studies are needed to test the clinical utility of the signature for effective treatment strategies and personalized therapies of LUSC.

## Supplementary Information

Supplementary Information.

## Data Availability

Raw RNA sequence data that support the findings of this study are available from the Gene Expression Omnibus (http://www.ncbi.nlm.nih.gov/geo/) or TCGA (https://www.cancer.gov/about-nci/organization/ccg/research/structural-genomics/tcga), respectively.

## Abbreviations

AUC: Area under the ROC curve
BP: Biological process
CC: Cellular component
DEGs: Differentially expressed genes
GEO: Gene Expression Omnibus
GO: Gene Ontology
GSEA: Gene Set Enrichment Analysis
ICIs: Immune checkpoint inhibitors
ICMs: Immune checkpoint molecules
KEGG: Kyoto Encyclopedia of Genes
KRT: Keratin
LASSO: Least absolute shrinkage and selection operator
LUAD: Lung adenocarcinoma
LUSC: Lung squamous cell carcinoma
MF: Molecular function
NES: Normalized enrichment scores
OS: Overall survival
PPI: Protein-protein interaction
ROC: Receiver operating characteristic
TCGA: The Cancer Genome Atlas
TME: Tumor microenvironment
