# Supplementary material for: A 13-gene signature to predict the prognosis and immunotherapy responses of lung squamous cell carcinoma
Source: Sci Rep. 2022 Aug 11;12:13646. doi: 10.1038/s41598-022-17735-6 (PMC9372044; doi:10.1038/s41598-022-17735-6)
Supplement: Supplementary file 1 — Supplementary Information. [file 41598_2022_17735_MOESM1_ESM.docx]

| **Gene symbol** | **Full name** | **Main function** |
| --- | --- | --- |
| **KLRC2** | Killer Cell Lectin Like Receptor C2 | Immune activating receptor involved in self-nonself discrimination |
| **CD1E** | CD1e Molecule | T-cell surface glycoprotein CD1e, soluble binds diacetylated lipids |
| **LIM2** | Lens Intrinsic Membrane Protein 2 | May contribute to cell junctional organization |
| **NPY** | Neuropeptide Y | Stimulation of food intake and modulation of circadian rhythm |
| **CDH12** | Cadherin 12 | Calcium-dependent cell adhesion proteins |
| **OTX2** | Orthodenticle Homeobox 2 | Involved in the development of the brain and the sense organs. |
| **ADRA1D** | Adrenoceptor Alpha 1D | Members of the adrenergic receptor group of G-protein-coupled receptors |
| **FGL1** | Fibrinogen Like 1 | Immune suppressive molecule that inhibits antigen-specific T-cell activation |
| **ZFP42** | ZFP42 Zinc Finger Protein | Involved in the reprogramming of X-chromosome inactivation |
| **GAGE2A** | G Antigen 2A | Cancer/Testis Antigen Family 4, Member 2;The role of GAGE in tumour metastasis is still controversial |
| **FGF4** | Fibroblast Growth Factor 4 | Regulation of embryonic development, cell proliferation, and cell differentiation |
| **F13A1** | Coagulation Factor XIII A Chain | Stabilizing the fibrin clot |
| **SAMD9L** | Sterile Alpha Motif Domain Containing 9 Like | May be involved in endosome fusion |

**Title:** **A 13-Gene Signature to Predict the Prognosis and Immunotherapy Responses of Lung Squamous Cell Carcinoma**

**Author list:** **Qin Yang, Han Gong, Jing Liu, Mao Ye, Wen Zou, Hui Li**

**Supplementary Table S1 Names and functions of the 13 prognostic genes.**
